# Adaptive Roles of *SSY1* and *SIR3* During Cycles of Growth and Starvation in *Saccharomyces cerevisiae* Populations Enriched for Quiescent or Nonquiescent Cells

**DOI:** 10.1534/g3.117.041749

**Published:** 2017-04-21

**Authors:** Dominika M. Wloch-Salamon, Katarzyna Tomala, Dimitra Aggeli, Barbara Dunn

**Affiliations:** * Institute of Environmental Sciences, Jagiellonian University, Krakow 30-387, Poland; † Department of Genetics, Stanford University, California 94305-5120

**Keywords:** *SSY1*, *SIR3*, SPS pathway, quiescence, evolution

## Abstract

Over its evolutionary history, *Saccharomyces cerevisiae* has evolved to be well-adapted to fluctuating nutrient availability. In the presence of sufficient nutrients, yeast cells continue to proliferate, but upon starvation haploid yeast cells enter stationary phase and differentiate into nonquiescent (NQ) and quiescent (Q) cells. Q cells survive stress better than NQ cells and show greater viability when nutrient-rich conditions are restored. To investigate the genes that may be involved in the differentiation of Q and NQ cells, we serially propagated yeast populations that were enriched for either only Q or only NQ cell types over many repeated growth–starvation cycles. After 30 cycles (equivalent to 300 generations), each enriched population produced a higher proportion of the enriched cell type compared to the starting population, suggestive of adaptive change. We also observed differences in each population’s fitness suggesting possible tradeoffs: clones from NQ lines were better adapted to logarithmic growth, while clones from Q lines were better adapted to starvation. Whole-genome sequencing of clones from Q- and NQ-enriched lines revealed mutations in genes involved in the stress response and survival in limiting nutrients (*ECM21*, *RSP5*, *MSN1*, *SIR4*, and *IRA2*) in both Q and NQ lines, but also differences between the two lines: NQ line clones had recurrent independent mutations affecting the Ssy1p-Ptr3p-Ssy5p (SPS) amino acid sensing pathway, while Q line clones had recurrent, independent mutations in *SIR3* and *FAS1*. Our results suggest that both sets of enriched-cell type lines responded to common, as well as distinct, selective pressures.

Organisms live in changing environments, and thus evolutionary adaptation to varying conditions is crucial for an organism’s long-term survival. Some environmental stressors may be rare while others may occur more frequently, even on a regular basis, such as circadian cycles. Such changes include fluctuation in temperature or nutrient levels (Tagkopoulos *et al.* 2008; Van der Linden *et al.* 2010), and seasonality is an ubiquitous driver of fluctuating selection in organisms with generation times of a month or less (Messer *et al.* 2016). A few mechanisms have been identified that can help organisms to prepare for recurring stressors (Dhar *et al.* 2013), for example, bet hedging, which results in expression of different sets of genes (or different levels of gene expression) within different subgroups of cells in the population, thereby generating phenotypic heterogeneity within an otherwise isogenic population. This strategy has been shown to increase the long-term fitness of yeast grown with variable application of either heat shock, “diauxic lag” phase duration, or utilization of different carbon sources (Levy *et al.* 2012; New *et al.* 2014; Wang *et al.* 2015). Another mechanism is adaptive anticipation, where an organism uses the information of the present environment to preadapt in the anticipation of the forthcoming changes. Physiological adaptive anticipation in single-cell organisms (including yeast) is well-documented and is becoming a new paradigm for microbiology (Mitchell *et al.* 2009, 2015; Brunke and Hube 2014; Siegal 2015; Yona *et al.* 2015).

In response to starvation for one or more nutrients, a fraction of the cells in a stationary yeast population exit the mitotic cycle and become Q, a state physiologically similar to that seen in higher eukaryotic G0 cells (Gray *et al.* 2004; Valcourt *et al.* 2012). The organization of a Q cell’s internal structures and genome is very different from that of a proliferating, NQ cell’s; there is an increase in storage carbohydrates and stress protectants such as glycogen and trehalose, increased width of the cell wall (Aragon *et al.* 2008), sequestration of proteins (Suresh *et al.* 2015), telomere clustering (Guidi *et al.* 2015; Rutledge *et al.* 2015; Laporte *et al.* 2016), and global transcriptional shutoff (McKnight *et al.* 2015; Young *et al.* 2017). These changes are programmed, energy dependent, and are considered physiologically adaptive during stress (Smets *et al.* 2010; Klosinska *et al.* 2011; De Virgilio 2012). Most of the transcriptional changes associated with transition to Q state are simply correlated with slower growth during starvation, and as such are not specific for the Q state (Valcourt *et al.* 2012). However, a set of Q-specific core genes, showing altered transcription rates during starvation that are independent of any growth rate-associated patterns of expression, has been previously identified. For example, increased transcription was detected for genes involved in membrane lipid biosynthesis, protein modification, response to toxins, and metal ion transport, while decreased transcription was found for genes related to cytokinesis, chromosome organization and biogenesis, and organization of the nuclear pore complex (Klosinska *et al.* 2011). Still, the most significant property of yeast Q cells, relative to proliferating NQ cells, is their ability to maintain viability over long periods of time during the growth-arrested phase, and to resume mitotic growth once growth-promoting conditions are restored (Gray *et al.* 2004). Transition to Q cells may be triggered by early signals of nutrient depletion, so that the population contains individuals in different physiological states with associated transcriptional profiles. Production of a stable fraction of Q cells is characteristic for yeast strains. A population-level bet hedging strategy could result in subgroups of Q cells and NQ cells, which may increase the chances of that population’s survival in variable environmental conditions (Wloch-Salamon *et al.* 2013; Wloch-Salamon 2014; Honigberg 2016).

The extracellular concentration of nutrients available for free-living fungi can change by many orders of magnitude during the growth of a population over time, and even during the lifespan of a single cell. Micro-organisms cope with such changes in part by controlling the rates of uptake of these molecules, by tuning their ability to sense them, and by transcriptional induction of relevant permease genes. In *Saccharomyces cerevisiae*, distinct mechanisms have evolved to sense the extracellular presence of carbon sources (such as glucose) and nitrogen sources (including amino acids) (Ozcan *et al.* 1996; Didion *et al.* 1998). *S. cerevisiae* appears to have adapted to a wide range of nutrient availability by maintaining at least 15 different amino acid permease genes and at least 17 sugar transporter genes (Gaber *et al.* 2003). An extreme adaptation to scarcity of nutrients is manifested in diploids by the production of spores, and in haploids by the production of Q cells.

In this study, we set out to identify genetic mechanisms involved in the regulation of Q cell formation. To this end, we repeatedly subjected populations of haploid *S. cerevisiae* cells to two sequential environmental conditions: (1) feast, under which adapted cells are expected to divide as quickly and efficiently as possible, and (2) famine, where some cells in the population transition to nondividing Q cells, which are able to survive long periods of starvation and thus are crucial to the long-term survival of the population. After each cycle, we enriched our experimental lines for either Q or NQ cells, exploiting the fact that Q cells are more dense than NQ cells (Allen *et al.* 2004), and repeated the process for 30 such cycles, which we determined was equivalent to ∼300 generations (cell doublings). For one set of lines, only Q cells were repeatedly propagated, while for the other set of lines, only NQ cells were propagated. The NQ line populations are expected to be enriched in cells that do better in the nutrient-rich condition, as they ignore environmental signals of nutrient depletion and do not enter the Q program. By contrast, the Q line populations are expected to be enriched in cells that do better under famine, detecting and/or anticipating upcoming nutrient scarcity, since repeated harvesting of only Q cells should reward cells that enter the Q cell program. For the Q-enriched population, we presume that fitness advantages during logarithmic growth become less important overall, because we assume that only Q cells make it to the next cycle. Because the populations were grown and then starved on the same rich medium (YPD plates) the change in environment was not acute, but instead was gradual. This may allow cells, when reaching early stationary phase, to naturally anticipate signals of the consecutive changes, and respond by differentiating into Q or NQ cells. After the 30 successive cycles of cell type enrichment, growth, starvation, and separation, we isolated several single colonies from the final populations for whole-genome sequencing. These 30-cycle clones (C30) were also used to investigate possible trade-offs in their fitness under conditions differing from those in which they were selectively enriched.

During the course of the experiment, we observed that populations produced a significantly increased fraction of the cell type (Q or NQ) for which they were enriched, which was also true for the C30 clones. Analysis of whole-genome sequences of the C30 clones allowed us to identify potentially Q- or NQ-promoting mutations. Interestingly, NQ cell enrichment in our conditions may be enhanced by disabling the SPS signaling pathway, as *SSY1* was frequently mutated in NQ lines but not in Q lines. In Q lines, we found repeated *SIR3* and *FAS1* mutations. We also found genes independently mutated in both lines, including *ECM21*, *RSP5*, *MSN1*, *SIR4*, and *IRA2*. Our results reveal distinct patterns of molecular adaptation for increased production of either Q or NQ cells.

## Materials and Methods

### Strains, media, and cycles of growth and starvation (G-S)

**Figure 1:**
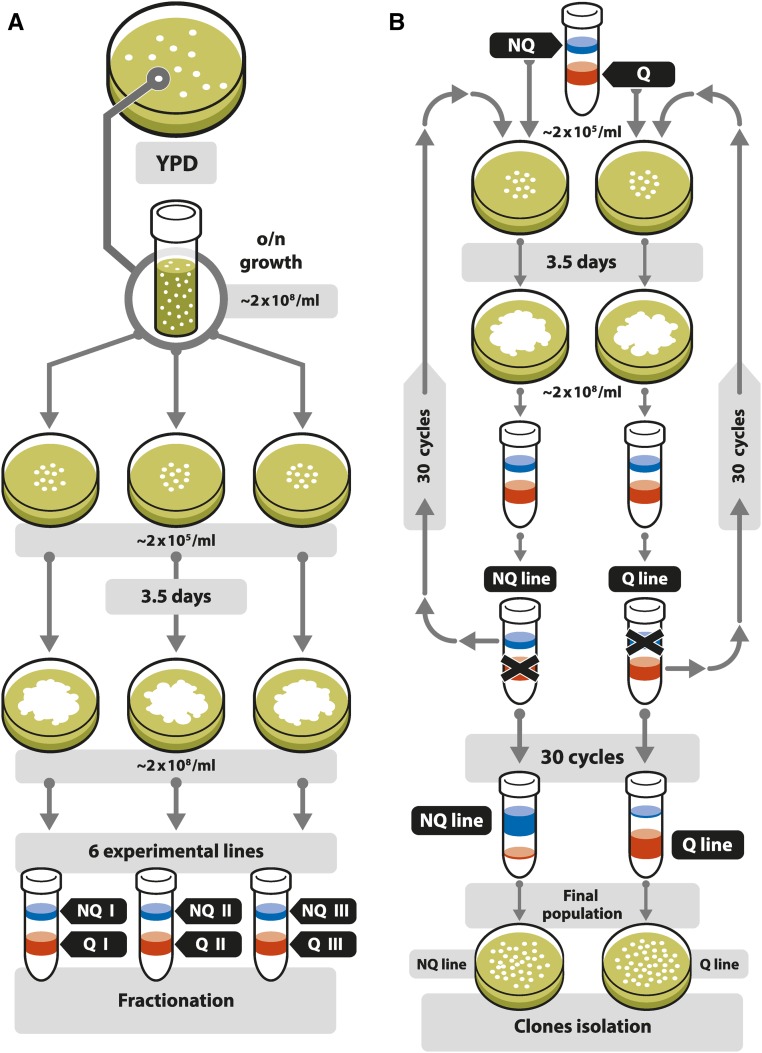
(A) Experimental design and (B) procedure shown for the separation of NQ and Q line populations. (A) We started the experiment with one clone, which was grown to stationary phase. This population was then diluted and plated onto three plates. The initial density of each population on the plate was ∼2 × 10^5^/ml. Plates were incubated for 3.5 d and all the cells were harvested. Each plate population was separately fractionated in Percoll solution. From each of the three plates we obtained two distinct fractions of cells that were founders of the six experimental lines. NQ, lines of NQ cell selection, Q, lines of Q cell selection. (B) Continuation of experiment shown via the example of one NQ and one Q line. We progressed the experiment by using only NQ or only Q cells from fractionated cultures, diluted them, and plated at an initial density of ∼2 × 10^5^/ml. Plates were incubated for 3.5 d, then cells were harvested, and fractionated. For the NQ lines we propagated only NQ cells, for Q lines only Q cells. We carried out the experiment for 30 cycles of growth and starvation. From the final fractionation, we isolated 15–16 clones from each experimental population for further analysis. NQ, nonquiescent; o/e, overnight; Q, quiescent.

We used a derivative of the laboratory haploid strain s288C (Mat α, *ura3*::KanMX4, *his3*Δ1 *leu2*Δ *lys2*Δ) [modified according to Cubillos *et al.* (2009)]. A single colony from a YPD plate was grown to stationary phase in 10 ml liquid YPD at 30° for 2 d (density of ∼2 × 10^8^ cell/ml). The culture was diluted 1:100 and three 100 μl aliquots were used to start replicate populations for the experiment by plating them as patches on 10 ml YPD small Petri plates (50 mm diameter), such that the initial density of the entire plate was ∼2 × 10^5^/ml. After 4 d of incubation at 28°, the entire patch was washed from the plate using 10 ml of sterile water, 4 ml of which was then fractionated (see below) in a Percoll gradient, to give two fractions: a more dense, lower fraction (Q) and a less dense, upper fraction (NQ). Each of the six resulting fractions (an NQ and a Q from each of the three replicates) was then used to found experimental lines NQ I–III, and Q I–III, respectively (Figure 1A). We used calibration curves, based on combined OD reads and Burker chamber cell counting for the NQ and Q lines, to plate 1 × 10^6^ cells onto a 10 ml YPD plate (for a final media density of 1 × 10^5^/ml), which was incubated for 3.5 d at 28°, at which point the cells had reached stationary phase (density ∼2 × 10^8^ cells/ml). The fractionation, followed by the YPD plating, was then repeated for 30 cycles. For all of the NQ experimental lines, we used only cells from the upper fractions (NQ cells) for each subsequent cycle, and likewise for the Q lines we selected only the lower fractions after each centrifugation (Q cells). Starting at cycle 10, we sampled aliquots of the populations from each fraction every five cycles, and stored them as frozen glycerol stocks at −80°. The total quantity of the Q and NQ cells in the populations was estimated by optical density.

### Fractionation and isolation of Q and NQ cells

We used a previously described method (Allen *et al.* 2006) to obtain the gradient for density fractionation. In brief, we mixed Percoll and 1.5 M NaCl, 9:1 v/v, and 7.5 ml of this solution was centrifuged in a swinging bucket rotor at 10,078 × *g* for 20 min. Then 4 ml of the culture washed from the plates (in 50 mM Tris, pH 7.5) were placed on top of the gradient and were centrifuged for 60 min at 417 × *g*. We carefully removed the upper (NQ) and lower (Q) cell fractions into separate tubes using pipettes. Based on OD, ∼10^6^ cells were transferred to a YPD plate of 10 ml, for an initial density of 10^5^ cells/ml (for the entire volume of the small Petri plate). In addition, at the beginning of the experiment, at the 10th and then every 5th cycle (that is 15th, 20th, 25th, and 30th), the quantity of each of the Q and NQ fractions in all experimental lines was assessed. All fractions were collected to separate tubes and then serially diluted. Based on specific density curves (separate for Q and NQ fraction) and spectrophotometric OD measurement, the population density was estimated.

### Refractionation

During the experiment we observed an increase in the quantity of all selected fractions in each of the experimental populations. That is, the NQ fractions from the NQ lines became thicker as did the Q fractions from the Q lines (Figure 1B). To determine if this phenotypic effect was heritable and still present in the populations derived from the evolved clones, we checked the quantity of NQ and Q fractions of the ancestral strain and 11 evolved clones. Latin numbers refer to the experiment (QI 1, 13; QII 5, 6; QIII 8, 9; NQI 3, 11; NQII 15; and NQIII 6, 7). Each of these clones and the ancestral clones were subjected to a single cycle of G-S prior to fractionation, similarly to as in the main experiment. It included growth on YPD plate for 3.5 d and single fractionation in a Percoll gradient. The number of cells in each of the isolated fractions was estimated based on OD measurements. This procedure was repeated three independent times for each of the clones.

### Phenotypic changes of evolved clones: maximum growth rate (MGR)

MGR of each C30 clone was estimated in liquid YPD medium culture. A frozen sample was transferred by a metal pin to fresh liquid YPD medium dispensed into 500 μl aliquots in 96-deep well plates and incubated without shaking for 3 d at 30°. Then, 2 μl of this stationary phase culture were transferred to 100 μl of fresh YPD liquid medium in a 96-well plate, which was then sealed. Plates were then placed into a Tecan instrument, incubated at 30° with agitation at 1250 rpm, and wide-band optical density (OD) readouts were acquired every 10 min with the Infinite m200 Microplate Reader (Tecan). The OD readouts were corrected for background effects, log-transformed, and used to determine the range of exponential growth. This was performed by finding Q and NQ limits of OD, between which the Pearson coefficient *r* was highest. Regressions were based on no less than 10 time points and had an *r* of 0.99. MGR (1/hr) was then estimated from the slope of the linear regression of the log OD *vs.* time. This experiment was repeated in triplicate for each C30 clone. One-way ANOVA and *post hoc* Tukey test were used for statistical analysis.

### Phenotypic changes of evolved clones: relative growth (RG)

Initially, reference and query strains were mixed in 1:1 proportions. The conditions used were exactly the same as for the cycles of G-S that were used during the experiment, except that instead of fractionation on a Percoll gradient we kept the populations in Percoll solution for 2 hr with no centrifugation or fractionation (the same amount of time that was required for centrifugation and sample collection during the experimental conditions), and then washed the cells using standard Tris buffer (as during the experimental procedure). The initial mixed populations were diluted to a density of 2 × 10^5^/ml to: (1) calculate their initial proportions and (2) start the actual G-S cycle. We applied two cycles of G-S, and then counted the fraction of cells of each competing strain by counting the number of fluorescent and nonfluorescent colonies on a YPD plate. Fitness as the RG rate on plates in competition with ancestral marked with YFP was measured assuming transitivity of fitness interactions. The YFP marker was amplified from genomic DNA from the BY4741-YFP.natR strain and integrated into the ancestral strain according to previously described protocol (DeLuna *et al.* 2008). The reference ancestral YFP-marked strain, 16 evolved experimental clones from NQ and Q lines (QI 1, 13; QII 5, 6, 12; QIII 8, 9, 11; NQI 11, 14; NQII 3, 15; and NQIII 6, 8, 11) and the unmarked, original ancestral strain, were separately grown overnight to stationary phase in liquid cultures. The reference strain was mixed with each of the query strains isostoichiometrically. Mixed cultures were diluted and ∼10^5^ cells were used to inoculate a plate (10 ml YPD) for the first round of G-S. After 3.5 d, the resulting large patches were harvested and incubated for 2 hr in Percoll solution. Then, ∼10^5^ cells were used to inoculate another plate (10 ml YPD) for the second round of G-S. After each round of G-S, the frequency of competing strains was estimated by plating dilutions to count CFU (using a transilluminator to distinguish colonies that were florescent). The fitness of each of the clones relative to the reference strain was computed as the ratio of the Malthusian parameters of both strains (Lenski 1991). At least three replicate competition experiments per strain were performed. Ancestral clones grew 6% (average RG = 1.06) faster than the YFP-marked derivative. We corrected for this difference by dividing growth of each evolved query clone by this number. General Linear Models: Nested ANOVA model was used: repetitions nested in clones that were nested in line. *Post hoc* Tukey test was used for statistical analysis.

### Phenotypic changes of evolved clones: regrowth after starvation

Clones derived from the final experimental cycle (C30) (Q I 1, 13; Q II 5, 6; Q III 8; NQ I 11; NQ II 3, 15; and NQ III 6, 11) were transferred from the frozen culture to fresh YPD agar medium using a metal pin. The colonies were incubated for 3 d. Then, a small quantity of a colony was transferred to liquid culture for overnight growth on a rotating wheel at 30°. The next day, five drops of 50 μl were put onto 25 ml fresh YPD plates such that the grown patches did not touch each other; this was done in triplicate. After 2, 4, 8, 15, and 22 d, the population density and viability were estimated. Each time, one patch from the plate was washed out using water. Cell density was estimated based on Coulter Counter results. Then we serially diluted the cultures and plated them on a fresh YPD plate. After 3 d of incubation at 30°, we counted CFU and estimated the fraction of the cells able to undertake mitotic divisions and grow to form a colony on the plate. Viability is expressed as the cell fraction of cells that were able to undertake mitotic division and form a colony in the fresh YPD plate.

### Plate-washing assay

The plate-washing assay was performed as described (Cullen and Sprague 2000). Ten microliters of the stored population derived from each of the experimental and ancestral clones were put on a YPD plate. Invasion was allowed to proceed for 3 d at 30°. Plates were washed with water to remove cells that did not invade the agar. Care was taken not to break the agar surface. Photos were taken right before and immediately after performing the washing assay. Colonies for which we observed visible intrusion into the agar surface were marked X when only part of the area of the original colony was visible, and XX when the whole area of the colony was visible (Supplemental Material, Figure S1).

### Whole-genome sequencing of evolved clones

We struck out the experimental populations from the three NQ (NQ I, NQ II, and NQ III) and three Q (Q I, Q II, and Q III) lines from the final cycle (C30) to single cells on YPD plates. We isolated two ancestral clones and a total of 47 clones from the NQ lines (16, 15, and 16 from NQ I, NQ II, and NQ III, respectively) and 47 clones from the Q lines (16, 16, and 15 from Q I, Q II, and Q III, respectively). Clones were grown separately in fresh liquid YPD in a 96-deep well plate. After 2 d of growth at 30°, we used the YeaStar Genomic DNA Kit (Zymoresearch) according to the provided protocol for isolation of total genomic DNA from each clone. We measured DNA concentrations using a Qubit fluorometer and the Quant-iT dsDNA HS kit (Invitrogen) (according to provided protocols), and prepared Nextera-based genomic libraries as previously described (Kryazhimskiy *et al.* 2014). These 96 (94 evolved and two ancestral clones) DNA libraries were mixed together and sequenced on a single flow-cell lane on an Illumina HiSequation 2000 to generate paired-end, 100 bp reads.

### Sequencing data analysis: counting SNV in whole-genome sequences

We used two independent methods to analyze whole-genome sequences. For the first method, obtained sequences were mapped to the reference genome (R64-1-1; RefSeq assembly accession: GCF 000146045.2) using bowtie2 software (Langmead and Salzberg 2012) with option very sensitive. Obtained SAM files were transformed to BAM, sorted, and indexed using samtools (for more information go to http://www.htslib.org/doc/samtools-0.1.19.html). Optical duplicates were deleted using samtools rmdup. Samtools mpileup was used to look for the SNV (with the following settings: -u, and –q20). The resulting file was transformed to VCF format (using bcftools view). Variant filtering was done with a custom Python script. Variants with the following setting were qualified for further analysis: not present in the ancestral (t02) sequence, QUAL parameter (in VCF file) was ≥30, coverage was >9, present in ≥85% of all good quality reads. Coverage and percent of variant confirming reads were extracted from the DP4 field of the VCF file. For annotation, we used software annotate-0.1 (http://depts.washington.edu/sfields/software/annotate/). For the second method, genomic analysis was done using CLC-Genomic Workbench 7.5 software. Unmapped postfilter, paired-end reads as FASTQ files provided by the Stanford Sequencing Centre were downloaded directly to the CLC GW software. We mapped two of the ancestral clones (t01 and t02) and 94 evolved, C30 clones to the previously downloaded reference sequence: *S. cerevisiae*. *EF4*. *68*. *dna*. *toplevel* (*Genome*). No masking was applied to the reference genome. Mapping was random with the following parameters set: mismatch cost 2, insertion and deletion costs 3, length of fraction 0.5, and similarity of the fraction 0.8. The mapped sequences of clone t01 were filtered against known variation in the mapped sequences of the t02 clone, which was treated as the reference. We only considered variation supported by >85% of the mapping reads. We mapped whole-genome sequences of the 94 evolved clones to the reference genome. Then, we checked basic variant detection using the following parameters: ignore broken pairs, ploidy: 2; minimum coverage: 5; minimum count: 2; minimum frequency: 50%. Detected single nucleotide polymorphisms (SNPs) were filtered against previously found variations in clone t02. Variations found in each of the clones (ignoring broken pairs and nonspecific matches) were checked for amino acid change in the annotated genes. Each of the SNPs was confirmed by visual inspection in ∼50% of the cases of the aligned reads, and then annotated as to whether they were in coding or noncoding regions.

### Construction of the mutation phylogenetic tree

A fasta file was generated for all clones, and each clone was represented by a sequence, made out of all the variant positions present across all clones. The file also had an entry for the reference sequence. The fasta sequences were aligned using the MUSCLE package (Edgar 2004). The aligned file was then converted to a format that can be read by programs in the PHYLIP software distributed by the author (Felsenstein 2005). The tree was generated by the programs dnapars, retree, and drawgram from the PHYLIP software and imported in illustrator CS6.

### Data availability

Strains and isolated clones are available upon request. Genome sequences have been deposited in SRA (NCBI) as BioProject: PRJNA383491, with accession number SRP104610. File S1 contains descriptions of all Supplemental files. The authors state that all data necessary for confirming the conclusions presented in the article are represented fully within the article.

## Results

### Experimental design

Experiments were performed using a derivative of the *S. cerevisiae* S288C laboratory strain (Matα, *ura3*::*KanMX4*, *his3*Δ, *leu2*Δ, *lys2*Δ) (Cubillos *et al.* 2009). A single colony of this strain was grown in liquid YPD media to stationary phase and the resulting population was separated into Q and NQ cells by density centrifugation on a Percoll gradient, with the upper (less dense) fraction consisting of NQ cells and the lower (more dense) fraction consisting of Q cells (Figure 1A) (Allen *et al.* 2006). We then serially propagated cells from only the Q cell layer, or from only the NQ layer, through 30 G-S cycles. Each cycle started by plating 100 µl of the culture, containing ∼10^6^ cells, as a “patch” onto a small Petri plate containing 10 ml of YPD agar medium (2% glucose), yielding an initial population density of 10^5^/ml for the entire plate of medium. After 3.5 d of growth at 28°, after the point where the cells had already reached stationary phase and were experiencing nutrient deprivation (density of ∼2 × 10^8^/ml for the whole plate) (Allen *et al.* 2006; Davidson *et al.* 2011), Q and NQ cells were separated by centrifugation, and the relevant fraction (Q or NQ) was diluted and ∼10^6^ cells plated again to give 10^5^ cells/ml (for the 10 ml YPD agar plate) to start the next cycle. In three of the experimental lines (named NQ I, NQ II, and NQ III) only NQ cells were serially propagated, while in the other three lines (Q I, Q II, and Q III) only the Q cells were serially propagated (Figure 1B). Each of the six experimental lines were subjected to 30 of these 3.5-d G-S cycles (Figure 1B). After the 30th cycle (∼120 d), 15–16 clones were isolated from each of the experimental lines (a total of 47 from NQ lines and 47 from Q lines) and were whole-genome sequenced to identify mutations that might have been selected by the one selection regime or the other. These clones (all or only chosen ones) were in addition checked for phenotypic traits: MGR, survival during G-S cycles, survival in starvation, and invasive growth.

### Increase of the enriched cell type proportion in the experimental populations

**Figure 2:**
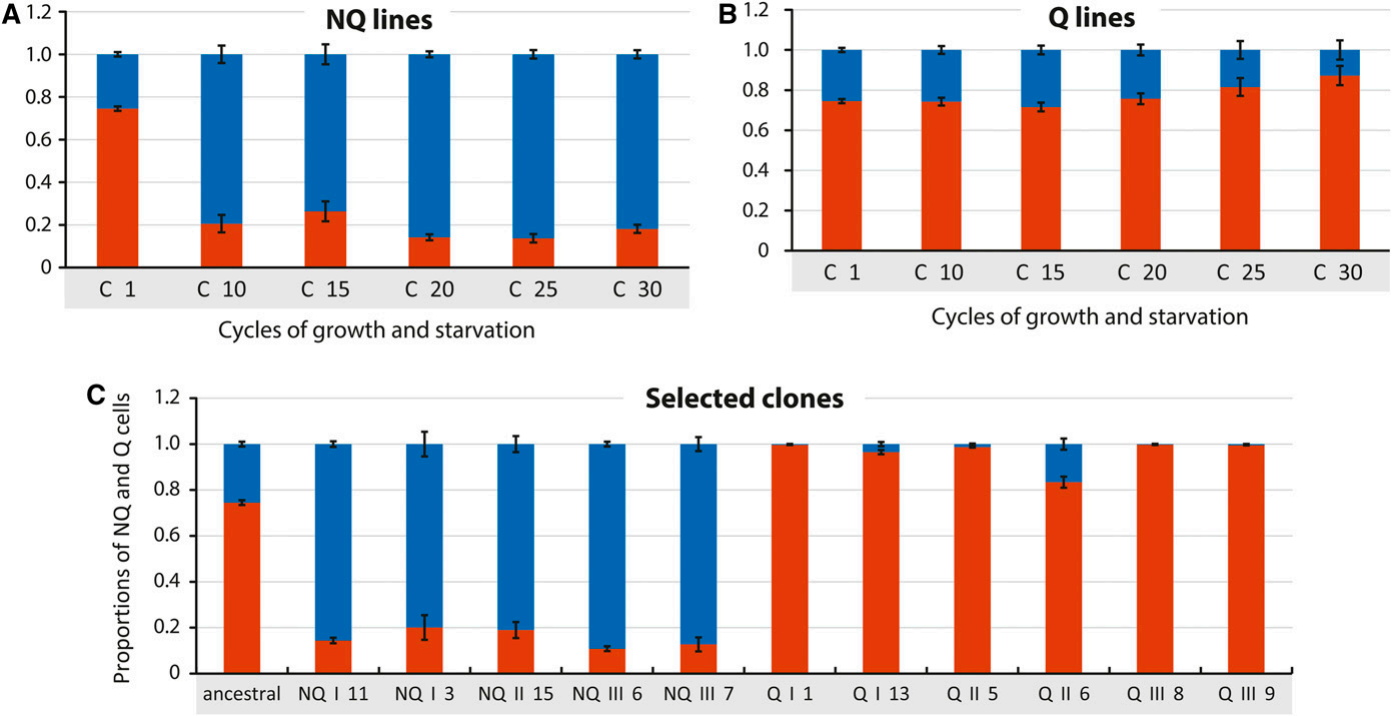
Average proportions of the quiescent (Q) cells (red bars) and nonquiescent (NQ) cells (blue bars) in the populations at the selected experimental cycles of growth and starvation in: (A) NQ lines (average of I–III) where only NQ cells were selected for following cycles of growth and starvation, and (B) Q lines (average I–III) where only Q cells were selected for the following cycle. (C) Average proportions of the quiescent (Q) cells (red bars) and nonquiescent (NQ) cells (blue bars) after one cycle of growth and starvation in the populations derived from the clones isolated after 30 cycles, at the end of the experiment, and in the ancestral strain (Figure 1B).

To determine whether serial enrichment of a particular cell fraction resulted in a stably increased production of such cells within the population, we measured the relative proportions of each cell type (on a population basis for each of the enriched lines) throughout the experiment. We observed significant increases in the relative proportion of each serially enriched cell type (Figure 2, A and B). The ancestral population, prior to the serial cell type enrichment cycles, had a NQ cell proportion typical of the S288C strain of 25% ± 1% and a corresponding Q cell proportion of 75% ± 1%. After 30 G-S cycles with serial enrichment for NQ cells, the proportion of NQ cells (taking the average of all three NQ lines) increased to 82% ± 2%, and the proportion of Q cells correspondingly decreased to 18% (Figure 2A). The percentage of NQ cells for the individual NQ I, NQ II, and NQ III lines, after 30 cycles, was 87, 79, and 80%, respectively. Likewise, after 30 G-S cycles, but with serial enrichment for Q cells, the percentage of Q cells (average of all Q lines) increased to 87% ± 5%, while NQ cells correspondingly decreased to 13% (Figure 2B). The 30-cycle Q percentages of the individual Q I, Q II, and Q III lines were 91, 76, and 95%, respectively. The Q II line, which acquired a mutation in *MMS2* and a mutator phenotype (explained below), showed no significant change in cell type proportions during the 30-cycle experiment.

To determine whether the changes in cell type proportions displayed by the enriched populations were also reflected by individual cells taken from the populations, we chose 11 single colonies from the six final (30th cycle) populations, and one colony from the ancestral preenrichment parent strain, and measured the fractions of each cell type that they generate under the experimental conditions. We performed one cycle of growth, starvation, and fractionation, identical to the experimental serial G-S enrichment cycles (Figure 1B) in triplicate for each of the chosen clones. The average proportion of NQ cells for all NQ lines was 84% ± 4%; the average for each line was: NQ I 88.9% ± 2.2%; NQ II 82.9% ± 2.2%; and NQ III 89.0% ± 8.9%. By contrast, the average proportion of Q cells in all Q lines was 96% ± 6%, and the average for each line was: Q I 98.59% ±7.9%; Q II 89.54% ± 6.6%; and Q III 99.61% ± 4% (Figure 2C).

### Adaptations to a specific environment results in trade-offs in different environments

We hypothesized that there may be fitness trade-offs due to adaptation to the specific conditions (Kassen 2002; Bono *et al.* 2016) that were used in our experiment, similar to previous findings (Wenger *et al.* 2011). To test this hypothesis, we determined the fitness of the ancestral clone and clones from the NQ and Q experimental lines in three different conditions: (1) Growth in YPD medium, with the presence of sufficient glucose (2%), *i.e.*, a condition to which clones producing increased numbers of constantly dividing NQ cells may be better adapted; (2) a fluctuating environment, consisting of two alternating G-S cycles, without separation and selection of cells (in this changing environment, the ancestral, “generalist” cells might be better adapted than either of the enriched, evolved “specialist” populations from Q or NQ lines); and (3) viability after long-term starvation and regrowth ability, a condition under which clones producing increased numbers of Q cells might be better adapted.

**Figure 3:**
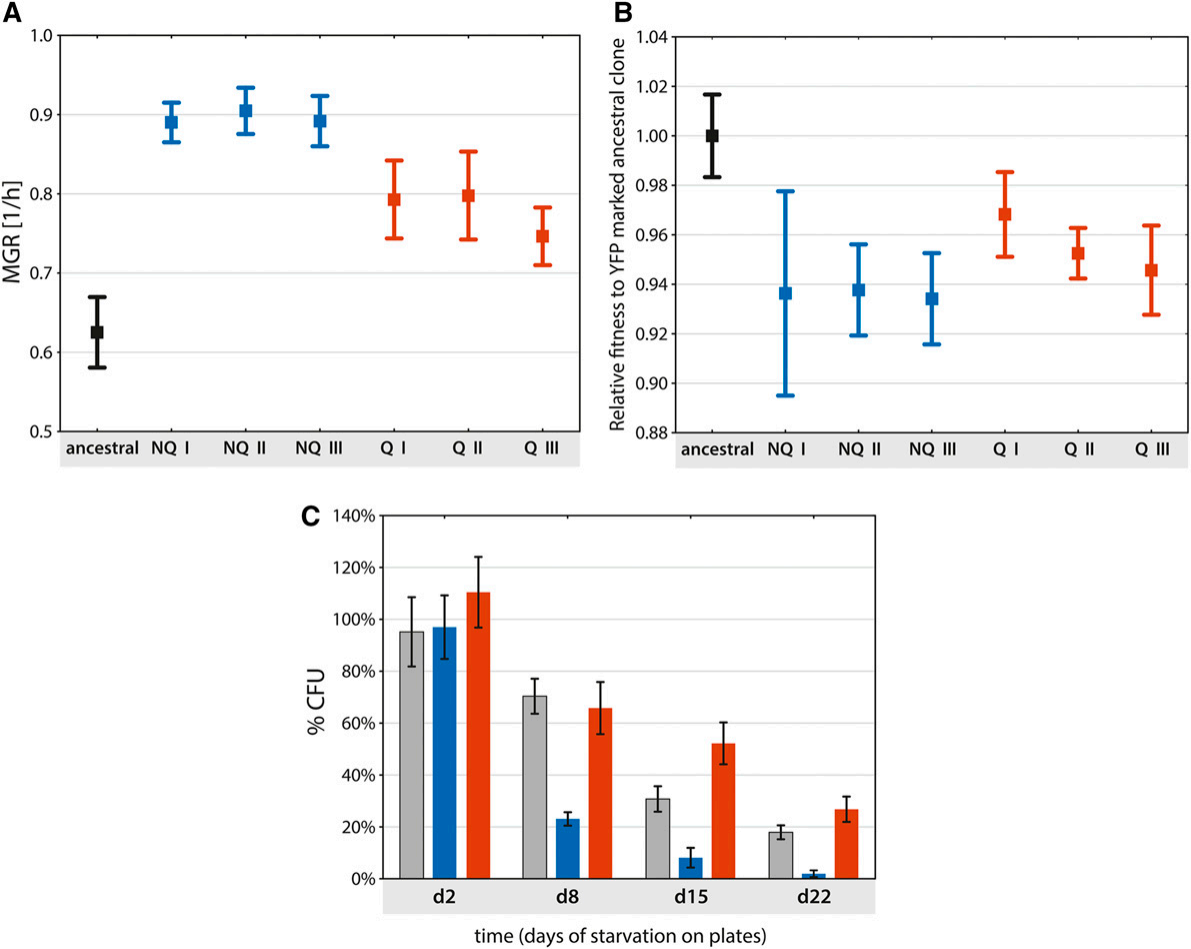
Test of trade-offs in fitness of evolved clones in three different environmental conditions. Each clone was measured at least three times. Error bars represent 95% C.I. (A) average maximum growth rate (MGR) of the ancestral clone and all clones from the final populations of each experimental line; NQ I: 16 clones, NQ II: 15 clones; NQ III: 16 clones; Q I: 16 clones; Q II: 13 clones; and Q III: 15 clones. (B) Fitness of the 16 selected clones from ancestral and experimental lines NQ I–III (NQI 11 and 14; NQII 3 and 15; NQIII 6, 8, 11, and 12; QI 1 and 13; QII 5, 6, and 12; QIII 8, 9, and 11) relative to yellow fluorescent protein (YFP)-marked ancestral strain. The result of the competition of ancestral and YFP-marked ancestral clone is standardized to one. (C) Colony-forming capacity of starved cultures determined by plating assay. Values are expressed as the percentage of colony forming units (CFU) to the total number of cells in the population. Gray bar represents ancestral population, blue and red stand for NQ and Q, respectively. NQ, nonquiescent; Q, quiescent.

Condition (1): We used MGR, measuring each evolved clones’ individual growth rate during logarithmic growth, as our assay. Division time (1/hr) measured in liquid YPD culture is an approximation for the early stage of each experimental G-S cycle, when cells are actively growing on solid YPD medium (Figure 1B). Under these conditions, we observed some differences between the lines (Figure 3A): one-way ANOVA showed differences in MGR between lines (*F* = 17.61, d.f. = 6, *P* < 0.0001), yet there was no significant difference between the NQ (I–III) and the Q (I–III) lines. Instead, a significant difference was observed between the ancestral and the evolved clones (*post hoc* Tukey test). All evolved populations statistically increased their MGR, with NQ lines having the highest MGR (Figure 3A). The MGR of the average of all (15–16) experimental clones from each experimental line (with ancestral MGR set as (1) was as follows: NQ cells: MGR_NQ I_ = 0.89 ± 0.05; MGR_NQ II_ = 0.90 ± 0.05; and MGR_NQ III_ = 0.89 ± 0.06; and Q cells: MGR_Q I_ = 0.79 ± 0.08; MGR_Q II_ = 0.80 ± 0.1; and MGR_Q III_ = 0.75 ± 0.06.

Condition (2): To assess the evolved clones’ adaptation to a changing environment, we measured the RG rate of 16 selected evolved experimental clones from NQ and Q lines and ancestral clones (“query” strains) in competition with a YFP-tagged version of the ancestral strain (the “reference” strain) on plates during two G-S cycles (Figure 3B). Nested ANOVA showed significant difference between lines (ancestral, NQ, and Q): *F*_lines_ = 9.57; d.f. = 6; *P* = 0.003; however, there was no difference between the clones within each line and within their replicates: *F*_clones_ = 1.25, d.f. = 12, *P* = 0.39, *F*_repetition_ = 0.89, d.f. = 34, *P* = 0.63). A *post hoc* Tukey test showed a significant difference in RG between the ancestral line (RG_ancestral_ = 1.00 ± SD = 0.01) and all NQ and Q II–III lines; however, a *post hoc* Tukey test showed that average RG from clones from the Q I line (RG_Q I_ = 1.02 ± 0.02) did not differ from the ancestral clone’s RG rate (Figure 3B). Though not significantly different, the average RG rate of Q clones showed higher fitness by 2% compared to the average of all the NQ line clones (RG_allNQlines_ = 0.99 ± 0.03 and RG_allQlines_ = 1.01 ± 0.02, respectively) (Figure 3B).

Condition (3): We examined the clonal population’s ability to regrow after starvation on YPD plates. We used two clones from the ancestral population, t01, and t02 (derived from the same single colony population that was used to start the whole experiment), and 10 clones from the experimental lines. From each clone, we derived populations that were checked for ability to regrow after 2, 8, 15, and 22 d of starvation on YPD plates. Analysis of GLM nested ANOVA showed significant differences between days (not nested) and lines (ancestral, Q, and NQ), but no difference between clones within lines (*F*_days_ = 166.210, d.f. = 3, *P* < 0.001; *F*_lines_ = 16.22, d.f. = 6, *P* < 0.001; and *F*_clones_ = 0.479, d.f. = 4, *P* = 0.75). Differences in viability, measured as the percentage of the entire population that was still able to reenter mitosis, divide, and produce a colony, became statistically significantly different among lines after 8 d of starvation with the Q lines showing a significant advantage. In this condition, clones derived from Q lines were fitter than either the ancestral or NQ line-derived clones (Figure 3C). The difference became more pronounced with increasing time of starvation, as measured on days 15 and 22.

### Strains may have acquired adaptive mutations during the experiment

To determine whether selection for Q or NQ cells resulted in the enrichment of particular mutations that might predispose cells to form those particular cell types, we whole-genome sequenced t01, t02, and 94 evolved clones (15–16 from each of the three Q and three NQ lines). In total, across all the sequenced genomes of all clones, we found 138 (including 13 frameshifts) nonunique events of nonsynonymous SNV within gene coding regions (Table S1). The phylogenetic relationships among clones are shown in the (Figure S1). The presence of multiple mutations shared across different experimental lines is an unexpected result. We can hypothesize that: (1) it is possible that some of the shared mutations arose early in the experiment, before splitting to independent lines, and as such were spread within the experimental lines; (2) that some of these mutations were present in the initial culture because although we used what we believed was a single colony, there may have been more than one colony that was picked (if that was the case, then low frequency mutations would not have been detected when we sequenced just two clones from the ancestral population); and (3) there could have been unintended mis-labeling during the experiments [if so, we believe it is most probable (although we cannot be sure) that there was not any switching between Q and NQ lines]. Overall, we feel that because our choice of the presumably adaptive mutations is based on the fact that multiple mutations in a gene are present in several experimental clones and not that the clones belong to any specific line (except what is shared between NQ or Q line), that if mis-labeling occurred, it did not influence the choice of which mutations were adaptive.

One of the identified frameshift mutations was in the *MMS2* gene (Thr44fs, middle branch in the Figure S1). The *MMS2* gene is involved in error-free postreplicative repair (Broomfield *et al.* 1998) and, when inactivated, confers a mutator phenotype, significantly increasing the spontaneous mutation rate (note that *mms2* mutants do not display any characteristic mutational spectrum) (Xiao *et al.* 1999). As expected, the 22 clones that carry this particular *MMS2* mutation have acquired substantially more SNV than the rest of the clones, with an average of 15.7 mutations per clone as compared to only 4.9 mutations per clone for the remaining 72 clones. These 22 clones are excluded from the following analysis and discussion; however, their associated datasets are included in Table S1. After excluding clones with the *MMS2* mutation, we identified 50 SNV in 40 genes (nuclear genome only), including one dubious ORF, and one gene of unknown function (Table S1) from a total of 72 sequenced clones. In 32 of these genes, only a single mutation was observed, with these mutations being present in 1–16 clones. Because of the experimental set-up where we transferred a fraction of the populations to the next G-S cycle, we do expect that clones isolated from each line at the end of the experiment (C30) would share some of the SNV that arose during the experiment. That is indeed the case, and SNV was often shared among clones with common ancestry from the same experimental line (Figure S1 and Table S1).

**Figure 4:**
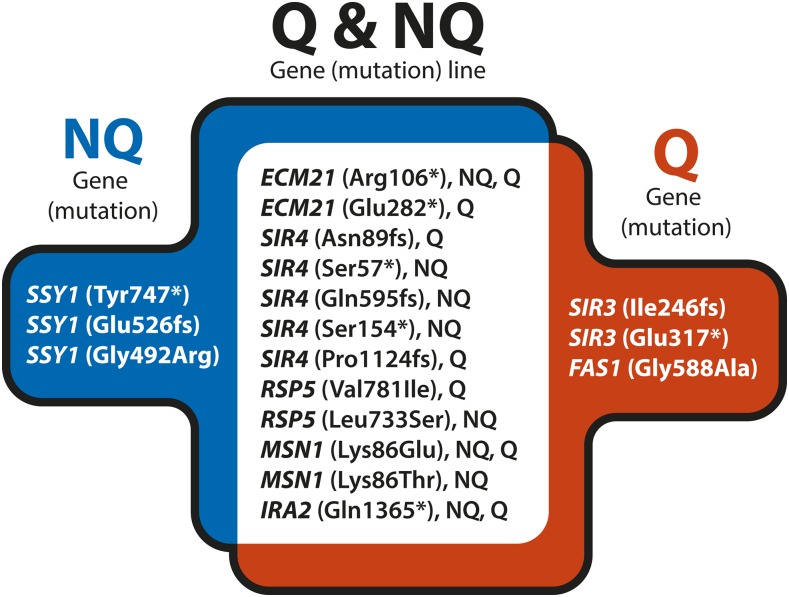
Presumably adaptive mutations identified in the whole-genome sequenced, nonmutator clones isolated from experimental Q and NQ lines on the 30th experimental cycle. A complete list of all identified mutations is in Table S1. NQ, nonquiescent; Q, quiescent.

To identify mutations that may have been selected by the experimental conditions, we focused on genes in which we observed multiple different mutations, either specific to only Q or NQ lines, or found among both Q and NQ lines. In NQ lines only, we observed multiple mutations in *SSY1*, while in Q lines only, we observed multiple mutations in *SIR3*. We also observed genes that were independently mutated multiple times in both Q and NQ lines (*MSN1*, *ECM21*, *SIR4*, and *RSP5*). We also focused on single mutations that were observed in many Q clones but no NQ clones (*FAS1*) or in all NQ clones and only three Q clones (*IRA2*). We interpret the mutations that arose in this group of eight genes as possibly adaptive to the growth and enrichment environments we performed (Figure 4).

### Adaptive mutations shared between NQ and Q lines

There are four genes in which we identified widespread multiple mutations across both the NQ and Q experimental lines (Figure S1 and Table S1). These are *MSN1*, *ECM21*, *RSP5* and *SIR4* (Figure 4). Two different missense mutations in *MSN1*, both affecting the same codon, are present among all non-*MMS2* clones: Lys86Thr is found in QI and QIII clones, and Lys86Glu is found in clones from all six lines (Q I–III and NQ I–III). These results suggest that these mutations arose in the beginning of the experiment before splitting of the lines. *MSN1* encodes a transcriptional activator of invertase and glucoamylase expression. *MSN1*-null mutants show either a decrease or complete absence of invasive growth, typical for haploids growing on solid agar in response to nitrogen and carbon limitation (Gagiano *et al.* 1999; Cullen and Sprague 2000). Because our experimental protocol involves a step where we collect cells for the next cycle by washing the cells off an agar plate, we performed a plate-washing test (see *Materials and Methods*) to determine the invasive growth phenotype for all 94 sequenced clones. Despite having a wild-type *MSN1* gene, the ancestor clones did not show an invasive growth phenotype; this was expected as the S288C strain background does not show invasive growth. The invasive growth phenotype was also absent from all 36 Q clones, even though they all had one of the two *MSN1* missense mutations, suggesting that these mutations do not increase invasive growth. However, we found that 33 NQ clones (out of a total of 36, all of which have the *MSN1* Lys86Glu mutation) do show an invasive growth phenotype (Figure S1 and Table S1). Thus, apparently, the *MSN1* mutations are not responsible for any changes in the invasive growth phenotype, and instead, the presence of other NQ line-specific mutations such as the SPS pathway genes (see *Discussion* below) may be conferring the invasive growth phenotype to the NQ clones. Interestingly, the three NQ clones that lack an invasive growth phenotype are the only clones (out of all sequenced clones) that contain a presumed inactivating frameshift mutation in the *RIM8* gene; null mutations of *RIM8* are known to decrease or abolish invasive growth (Madhani and Fink 1998), and thus this mutation may be responsible for the lack of invasive growth in these NQ clones. Overall, invasive growth among NQ cells suggests an expected phenotypic trait for cells in search of nutrients, as opposed to cells more prone to enter quiescence.

We also observed multiple mutations in *SIR4* and *ECM21* across both Q and NQ cell lines. We observed five mutations (Ser57*, Asn89fs, Ser154*, Gln595fs, and Pro1124fs) in the *SIR4* gene, which regulates *de novo* assembly of heterochromatin by scaffolding, nucleation, and anchoring (Kueng *et al.* 2012, 2013). Mutated sites are widespread along the entirety of this long gene (1358 aa). The first three mutations, all presumably inactivating since they are nonsense or frameshift, occur within the first domain (aa 1–270), which is responsible for yKu80 and DNA binding. The Gly595fs frameshift mutation leaves the above yKu80/DNA binding domain intact as well as a second Rap1 binding domain (aa 142–591) intact, although it is not known if a partial protein would be stable. The frameshift mutation Pro1124fs resides in the last domain, which is responsible for Sir4p dimerization and Sir3p and YKu70 binding (Kueng *et al.* 2012, 2013; Gartenberg and Smith 2016). Found among all lines, and shared by 67 clones, were two mutations in the *ECM21* gene. Both mutations introduce premature stop (positions Arg106* and Glu282*) before the PY-motif, which is necessary for interaction with Rsp5p (Lin *et al.* 2008).

### IRA2 mutation found in all NQ clones

An *IRA2* stop mutation, Gln1365*, is present in 39 clones that were derived mainly from NQ cell lines (the mutation is present in all 36 NQ clones, but in only three Q clones). It is possible that this mutation also arose at the beginning of the experiment, before the culture was split between the NQ and Q selection lines. Ira1 and Ira2 are Ras GTPase-activating proteins (GAP) that act as negative regulators of the Ras-cAMP signaling pathway by increasing the rate Ras proteins (encoded by *RAS1* and *RAS2*) hydrolyze GTP to GDP (Tanaka *et al.* 1990). Such mutations in the Ras/cAMP signaling pathway have been previously identified as adaptive under glucose-limited conditions (Lang *et al.* 2013; Chiotti *et al.* 2014). These mutations result in elevated glucose signaling, resulting in the cell’s misperception that glucose is present even when it is not (Smets *et al.* 2010).

### The amino acid signaling pathway (SPS) and endocytosis may play a role in the enrichment of NQ cell frequency

There are 22 mutated genes among the 36 non-*MMS2* NQ clones (Table S1). YeastMine GO analysis of this gene group reveals enrichment for the biological process of *response to amino acid* (GOID 0043200, Bonferroni corrected *P*-value = 0.05) based on the presence of three genes in the group, *SSY1*, *PTR3*, and *SSY5*, which together constitute the SPS amino acid sensing pathway (Didion *et al.* 1998). Another enriched process is *ubiquitin-dependent endocytosis* (GOID 0070086, Bonferroni corrected *P*-value = 0.04) based on the genes *ECM21*, *RSP5*, and *RIM8*. The most significantly enriched cellular components are the *plasma membrane part* (GOID 0044459, Bonferroni corrected *P*-value = 0.006), based on genes *PHO89*, *SSY1*, *RSP5*, *PTR3*, *RIM8*, *SSY5*, and *IRA2*; the *plasma membrane extrinsic component* (GOID 0019897, Bonferroni corrected *P*-value = 0.03) due to genes *RSP5*, *PTR3*, and *SSY5*; and the *plasma cytoplasmic side* (GOID 0009898, Bonferroni corrected *P*-value = 0.04) due to genes *RSP5*, *RIM8*, and *IRA2*.

We identified multiple mutations in the *SSY1* gene, all of which occurred only in clones derived from NQ lines (Figure 4, Table S1). The *SSY1* gene encodes a nontransporting nutrient sensor, and acts as the first step in the SPS pathway (Didion *et al.* 1998; Klasson *et al.* 1999) that senses extracellular amino acids (the following steps involve the *PTR3* and *SSY5* gene products, see above). All four of our observed *SSY1* mutations, Tyr747*, *853Tyr, Glu526fs, and Gly492Arg, presumably leave an intact cytoplasmic domain (Conrad *et al.* 2014). The other members of the SPS amino acid signaling pathway are mutated only in the NQ clones (*PTR3*: D263fs and *SSY5*: E155fs). Quite interestingly, we observed mutations related to SPS signaling and membrane receptor catabolism across all Q-enriched lines. In particular, point mutations in *ASI1*, *BRO1*, *DOA1*, and *RSP5* arise exclusively in Q lines (apart from a single instance in NQ lines, involving *RSP5*). It was shown that mutations affecting these genes restore amino acid uptake in SPS sensor-deficient strains (*ssy1leu2*). This allows for growth of cells that are otherwise unable to respond to extracellular amino acid concentrations, by compromising degradation pathways responsible for membrane receptor turnover (Forsberg *et al.* 2001). The expectation then would be that these mutants, which arose in clones from the Q lines, should result in upregulated degradation pathways. This mechanism is also suggested by studies linking degradation pathways to chronological lifespan in yeasts and other systems (Alvers *et al.* 2009; Takeda *et al.* 2010; Kruegel *et al.* 2011).

### Chromatin silencing role in populations with an elevated Q cell fraction

We found 20 genes mutated in the 36 non-*MMS2* clones from Q lines (Table S1). Yeast Mine GO analysis of this gene group showed enrichment in the cellular components of *transcriptional repressor complex* (GOID 0090568; 0017053, Bonferroni corrected *P*-value, respectively, = 0.001 and 0.003), due to the presence of genes *SIR3*, *SIR4*, and *BUR6* in the group, and enrichment in *cytoplasmic side of membrane* (GOID 0009898; Bonferroni corrected *P*-value = 0.02, genes: *RSP5*, *BUD2*, and *IRA2*).

We identified two mutations in the *SIR3* gene present in a total of 16 Q clones (Figure 4, Figure S1 and Table S1). Mutation E317* is present in clones from all three Q lines, suggesting that it might have been preexisting before splitting into experimental lines, or that it might be the result of unintentional mislabeling of clones (as explained before). An additional mutation in the *SIR3* gene is present within the QII line clones that carry the *MMS2* mutation. Both *SIR3* mutations (E317* and I246fs) leave the first *SIR3* domain, BAH (bromo-adjacent homology, aa 1–214), intact, which is crucial for chromatin repression. All 15 of the clones with *SIR3* E317* mutations have a preexisting *FAS1* missense mutation (G588A) that may have a synergistic effect. It has been suggested that the Fas1 “housekeeping” gene product (a subunit of fatty acid synthetase), in addition to its catalytic function, is required for the biosynthetic control of the yeast FAS complex (Wenz *et al.* 2001).

### Mutations in SIR2 and other possible adaptive mutations among the clones with MMS2 mutation (mutator lineage)

We cannot entirely exclude the possibility of additional adaptive mutations in the clones with the *MMS2* mutation (mutator clones). The presence of additional, including multiple, SNVs in the genes that we classified as possibly adaptive—such as *MSN1* (Ser200Phe and Arg186Trp) found in Q and NQ mutator clones, *SIR3* (Val653Phe) found only in Q mutator clones, and *SSY1* (*853Tyr) present only in NQ mutator clones—supports this hypothesis. There are two genes that acquired multiple SNVs, at least one of which is present within the mutator clones: *SIR2* (Leu332Pro, Cys469*, and Ser420*) and *KEL1* (Arg237Thr and Gln814*). All of the *SIR2* mutations are spread within NQ clones. This may reflect the unique role of Sir2p among sir complex genes in the asymmetric partitioning of oxidation-damaged proteins between mother and daughter cells (Aguilaniu *et al.* 2003). Furthermore, Sir2p is responsible for rDNA silencing (Smith *et al.* 1998), an activity that supports entry to quiescence. Mutations in *SIR2* and *SSY1* cooccur as in the cases of C469* in *SIR2*/G492R in *SSY1* and L332P in *SIR2*/*853Y in *SSY1* (the latter pair of mutations appears within the mutator line). This might suggest an epistatic relationship between these genes that confers fitness advantage among NQ cells.

Finally, there are five genes with multiple SNVs present only within mutator clones; *HSL7* (Val198Glu and Thr347fs), *EFM4* (Ser96Tyr and Ser21Pro), *SSK2* (Tyr852Ser and Leu853*), *WHI2* (Gly141* and Leu76fs), and *BRO1* (Asp228Tyr and Leu216Ile).

Two mutations, G141* and Leu76fs, that deactivate the *WHI2* gene are present within 26 mutator clones. The mutations leave intact the first out of five protein domains, similar to the mutations found previously in yeast evolving under nutrient-scarce conditions (Wenger *et al.* 2011; Lang *et al.* 2013). Whi2p is required for full activation of the general stress response and regulates growth during the diauxic shift. Null mutants have increased competitive fitness in YPD media. We also found two mutations in the *SSK2* gene (L853* and Y852S) in the mutator strains. Ssk2 is a kinase (MAPK) of the Hog1 mitogen activated pathway, activated in yeast by high osmolarity stress (Hersen *et al.* 2008). Although it is difficult to presume the adaptive nature of these mutations in strains that contain many other mutations simultaneously, the presence of these mutations (the mutations in the same genes as seen in non-*MMS2* clones, as well as the *WHI2* and *SSK2* mutations seen only in *MMS2* clones) is suggestive of an adaptive nature.

## Discussion

In this study, we sought to elucidate genetic mechanisms involved in the regulation of Q cell formation. Haploid yeast cells were sequentially subjected to two opposite environmental conditions of (1) feast, when cells should divide as quickly and efficiently as possible, and (2) famine, when transition to quiescence is crucial for survival. After successive enrichment for only one type of cells (either NQ or Q) that were used to found subsequent generations, we observed changes that indicated cell survival strategies differing from the ancestor. The enriched cell lines showed persistent and significant differences from the ancestor in proportions of Q and NQ cells, and also showed fitness trade-offs when grown in different environments. We found that the ancestral clone, a laboratory strain that presumably has been long adapted to a changing environment due to cyclic growth (serial transfer) on rich YPD-type medium, has higher fitness than either the NQ- or Q-enriched cell line clones in an assay monitoring growth through two feast–famine cycles; the ancestor thus retained its ability to both grow fast when nutrients are abundant, but also to be able to physiologically adapt to periods of starvation while nutrients are scarce. By contrast, clones from the NQ lines, which we found to be much more highly enriched in NQ cells than the ancestral strain, grow faster than either the ancestor or Q line cells in conditions when logarithmic growth rate is supported and there is no need to produce Q cells. Finally, clones from the Q experimental lines, which produce a slightly (but significantly) higher percentage of Q cells than the ancestor, outcompete the ancestral as well as NQ line clones in viability assays after prolonged starvation.

There are no easy-to-use markers for Q and NQ cells developed so far (Čáp *et al.* 2009; Rutledge *et al.* 2015; Laporte *et al.* 2016); however, separation of Q and NQ cells from the population can be achieved based on differences between the densities of NQ and Q cells (Allen *et al.* 2006; Davidson *et al.* 2011; Klosinska *et al.* 2011; Čáp *et al.* 2012), which is the method we used for our serial enrichment strategy. However, it is possible that to some extent we also selected or enriched for cells of various density or size characteristics not necessarily linked to their Q or NQ status. Although none of the genes we identified as possibly adaptive (Figure 4) were listed in the yeast GO annotation as *cell size control* regulator (GO:0008361), we cannot unequivocally exclude such a possibility, since the effects of gene mutations on cell size and density are not usually assessed, which also takes into account the fact that most clones acquired several mutations during the experiment.

### Increase of the NQ cell fraction may be a consequence of a dysregulated SPS signaling pathway

An increase in the production of NQ cells in the NQ lines was already visible after 10 cycles of G-S and did not change much after further cycles (Figure 2A). The most striking result from the whole-genome sequencing of our C30 serially enriched clones was the fact that multiple mutations in the *SSY1* gene (Tyr747*, Glu526fs, and Gly492Arg) were found in 32 of the 36 NQ clones (note that this does not include the *MMS2* mutator NQ clones that also have mutations in the *SSY1* gene, see Supplemental Material). The *SSY1* gene encodes a nontransporting nutrient sensor (Didion *et al.* 1998; Klasson *et al.* 1999), acting as the first step in the SPS pathway, during which the extracellular amino acid concentration results in conformational changes of the Ssy1p cytoplasmic domain, which signals to downstream targets (Conrad *et al.* 2014). In the presence of extracellular amino acids, the primary amino acid sensor Ssy1p is stabilized in a conformation that triggers intracellular signaling (Ljungdahl and Daignan-Fornier 2012). It has been previously observed that mutation of *SSY1* codon 382 from threonine to lysine (or arginine) results in constitutive signaling, showing that mutant Ssy1p can initiate signaling in the absence of extracellular signaling (Gaber *et al.* 2003; Poulsen *et al.* 2005, 2008). All the mutations we observed (Gly492Arg, Glu526fs, and Tyr747*, plus *853Tyr in the mutator clones) presumably leave an intact cytoplasmic domain (Conrad *et al.* 2014). This, similarly to previous discoveries (Gaber *et al.* 2003; Poulsen *et al.* 2005, 2008), could possibly result in constitutively active signaling in the absence of extracellular amino acids. In all the clones with *SSY1* mutations, the other genes from the SPS pathway were not mutated, which is also the condition for constitutive signaling found in previous research (Gaber *et al.* 2003). *PTR3* and *SSY5* were also mutated but in NQ clones with no mutation in *SSY1* (Table S1). In our experiments, an altered activity of the SPS pathway could be a mechanism that led to increased NQ cell production. In this rationale, constitutive activity of Ssy1p, the extracellular amino acid sensor, could lead cells to overestimate the amount of extracellular nutrients, which should hinder entry to quiescence. Alternatively, if the mutations are inactivating, downregulation of the pathway, *e.g.*, by inactivation of the downstream effector Ssy5p, increases replicative lifespan (Tsang *et al.* 2015); again, this is a trait that is expected to confer a fitness advantage among NQ cells.

Another gene that, when mutated, may confer advantage to NQ cells is *IRA2*; we observed a single frameshift (and thus possibly inactivating) mutation that was present in all 36 NQ clones, but in only three Q clones (Table S1). We presume that this mutation is more adaptive for the NQ than the Q cells. Inactivating mutations in *IRA2* (and its close homolog *IRA1*) are known to provide a fitness benefit in multiple contexts in glucose-limiting conditions (Wenger *et al.* 2011; Kvitek and Sherlock 2013; Chiotti *et al.* 2014; Venkataram *et al.* 2016) and can result in cells continuing to divide even in conditions of low glucose. Interestingly, *IRA2* had an elevated transcriptome level during glucose quiescence (Klosinska *et al.* 2011). Taken together, it seems quite plausible that the *IRA2* frameshift mutation may provide a fitness benefit for NQ-enriched lines, where uncontrolled growth during early starvation conditions may confer an advantage.

### Q cell advantage may arise from a possible role of SIR3 mutations

We observed a significant increase in the fraction of Q cells in two Q lines (Q I and Q III lines). Ultimately, Q line cells survive to the next experimental cycle only when they efficiently convert to the Q state. However, these cells also need to be able to reestablish cell division when the conditions improve. Both of these needs have to be taken into account when considering the possible role of the selected mutations. Present in all Q lines, two mutations in the *SIR3* gene (Ile246fs and E317*) introduce truncation after the BAH domain. Remarkably, the BAH domain alone has the binding properties of full-length Sir3p, suggesting that the domain represents an independent nucleosome-binding module (Connelly *et al.* 2006; Onishi *et al.* 2007). We hypothesize that the *SIR3* mutant product is still available for selective binding, which attracts the *SIR2-SIR4* complex. Thus, the chromatin silencing function is sustained. However, if the protein is truncated, it would seem that Sir3p dimerization through its extreme C-terminal wH (winged helix) domain, necessary for SIR-complex spreading along nucleosomes, might be impaired (Kueng *et al.* 2013). *SIR3* also has an important role in telomere hypercluster formation that extends chronological life span during quiescence. However, this is a rather slow process that reaches its plateau at ∼6–7 d of culture at 30° (Guidi *et al.* 2015). In addition, recent research shows that telomere hyperclustering is not required for cell survival in early quiescence (Laporte *et al.* 2016). Thus, for our experiments, where cells were starved for >3 d, we speculate that telomere hyperclusters may not have yet formed, and that this function of Sir3p may not be not adaptive during our short-term starvation conditions, although it is possible that it plays an early role that is adaptive in our conditions. Sir3p also plays a role in the epigenetic inheritance of silent chromatin in yeast (Motwani *et al.* 2012). Differentiation into distinct cell types involves establishing unique patterns of gene expression. Once established, epigenetic mechanisms operate to maintain these patterns as cells divide.

### Mutations shared among Q and NQ lines are important for the general stress response and nutrient acquisition

Invasive growth is believed to allow budding yeast to search for nutrients, as the elongated cells remain associated and in communication with each other, but also to anchor yeast colonies to the stable surface (Honigberg 2016). Thus, as the population proliferates, cells can invade solid agar media or move through static liquid media in search of carbon or nitrogen. The invasive growth test revealed an obvious distinction between NQ cells, in which the invasive phenotype was present, and Q cells, where this phenotype was completely absent (Figure S1). This was the case even for Q cells that were carrying the *MMS2* mutation, suggesting that other factors influence this phenotype. Interestingly, *rim8* mutation within a NQ lineage appeared to reduce invasiveness (Figure S1), consistent with a role of *RIM8* in invasive growth (Madhani and Fink 1998). Mutations, such as in *IRA2* (despite the predominant partitioning in NQ lines) and *MSN1* may directly reflect additional modes of adaptation in the growth media, nutrient depletion, and/or yeast harvesting from the agar plate.

### Conclusions

With this experimental set-up we wanted to begin to explore the regulation of the yeast Q state (Klosinska *et al.* 2011; Čáp *et al.* 2012; Honigberg 2016) and the growing interest in nonnatural, oscillating environment experimental design (Mitchell *et al.* 2015). Nutrient depletion causes yeast populations to produce Q cells at a certain frequency. By introducing selection and serial enrichment of Q or NQ cells, we uncovered pathways that appear to facilitate or hinder entry to quiescence, respectively. In the case of NQ-enriched cells, the presumed adaptive mutations we observed in the SPS signaling pathway may result in dysregulation of their adaptive anticipation mechanism and thus allow them to bypass transition to quiescence. In the case of Q-enriched cell populations, we speculate that because they were repeatedly subjected to >3.5 d of starvation, the deep reorganization of the internal cell structures such as (1) spatial confinement of enzymes, (2) mitochondrial and ER protein reorganization, and (3) loss of organelle contact sites might be not necessary or even a “waste of energy” (Suresh *et al.* 2015). In such a case it could be advantageous for cells to avoid it. All of the adaptations we observed for NQ- and Q-enriched populations seem to be costly and incur trade-offs in the other conditions. Our findings demonstrate the value of nonnatural dynamic perturbations in exposing hidden sensitivities of cellular regulatory networks.

## Supplementary Material

Supplemental material is available online at www.g3journal.org/lookup/suppl/doi:10.1534/g3.117.041749/-/DC1.
