# Supplementary figures and images for: Adaptive Roles of SSY1 and SIR3 During Cycles of Growth and Starvation in Saccharomyces cerevisiae Populations Enriched for Quiescent or Nonquiescent Cells
Source: G3 (Bethesda). 2017 Apr 21;7(6):1899–911. doi: 10.1534/g3.117.041749 (PMC5473767; doi:10.1534/g3.117.041749)

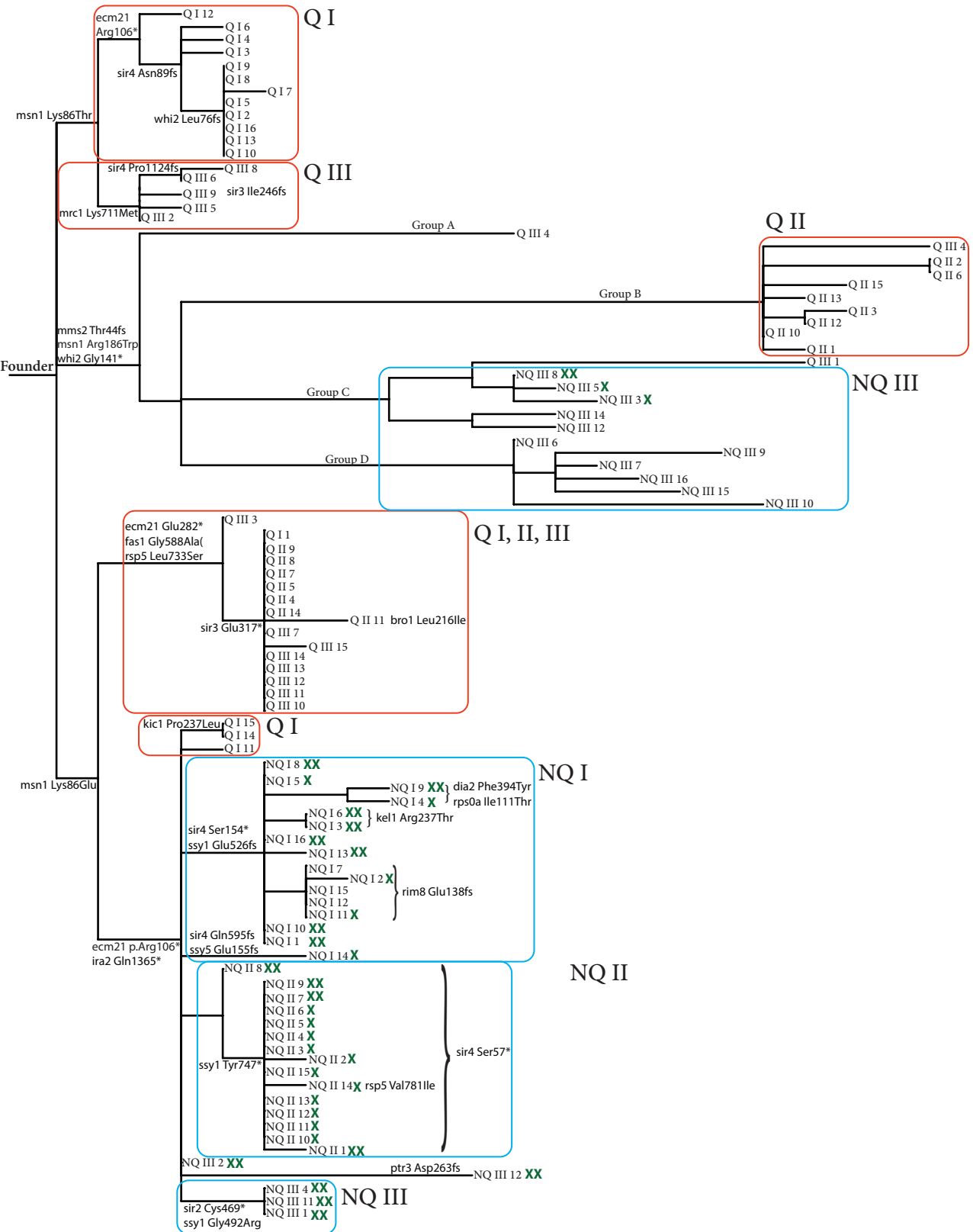

Supplement: Supplementary file 1 [file 1899FigureS1.pdf]
