# Supplementary material for: Adaptive Roles of SSY1 and SIR3 During Cycles of Growth and Starvation in Saccharomyces cerevisiae Populations Enriched for Quiescent or Nonquiescent Cells
Source: G3 (Bethesda). 2017 Apr 21;7(6):1899–911. doi: 10.1534/g3.117.041749 (PMC5473767; doi:10.1534/g3.117.041749)
Supplement: Supplementary file 3 [file 1899FileS1.docx]

**SUPPLEMENTARY MATERIALS**

File S1 contains detailed descriptions of all supplemental files.

Table S1. Summary of mutations found. Genes marked in red are genes with multiple mutations only in Q lines; Genes marked in blue are genes with multiple mutations only in NQ lines; genes marked in green are genes with multiple mutations in Q and NQ lines; Genes on orange background are present only in *mutator* clones (with *MMS2*  mutation). Presence of the mutation is marked as +. 0 means that mutation was absent in the clone. Mutator clones marked in red (with *MMS2* mutations).

Figure S1. Phylogenetic tree of the whole genome sequenced clones. The tree was constructed as follows: A fasta file was generated for all clones, and each clone was represented by a sequence, made out of all the variant positions present across all clones. The file had also an entry for the reference sequence, which was used as the outgroup root (noted as founder). The fasta sequences were aligned using the MUSCLE package (Edgar 2004). The aligned file was then converted to a format that can be read by programs in the PHYLIP software distributed by the author (Felsenstein 2005). The tree was generated by the programs dnapars, retree and drawgram from the PHYLIP software and imported in illustrator CS6. Clones from the same lines that group together are framed in red and blue for the Q and NQ lines, respectively. Genes with mutations that appear in a single clone are omitted for clarity, apart from the *SSY5* and *PDR3* mutations and the length of the branches reflect phylogenetic distance. For a complete list of mutations see supplemental table 1. Green XX and X stand for intensive and less intensive invasive growth tested by the washing test.
